# Animal movement on the hoof and on the cart and its implications for understanding exchange within the Indus Civilisation

**DOI:** 10.1038/s41598-023-50249-3

**Published:** 2024-01-02

**Authors:** C. A. Petrie, E. Lightfoot, P. J. Jones, J. R. Walker, B. T. Valentine, J. Krigbaum, P. le Roux, P. P. Joglekar, V. Shinde, R. N. Singh, T. C. O’Connell

**Affiliations:** 1 Department of Archaeology, University of Cambridge, Cambridge, UK; 2 Menzies Institute for Medical Research, University of Tasmania, Hobart, Australia; 3 Department of Anthropology, University of Florida, Gainesville, USA; 4 Department of Geological Sciences, University of Cape Town, Cape Town, South Africa; 5 Department of Archaeology, Deccan College, Pune, India; 6 Department of AIHC and Archaeology, Banaras Hindu University, Varanasi, India

**Keywords:** Archaeology, Stable isotope analysis

## Abstract

Movement of resources was essential to the survival and success of early complex societies. The sources and destinations of goods and the means of transportation – be it by boats, carts and/or foot – can often be inferred, but the logistics of these movements are inherently more difficult to ascertain. Here, we use strontium isotopic analysis to test hypotheses about the role of animal and animal-powered transport in medium and long-distance movement and exchange, using the Indus Civilization as a case study. Across the wide geographical spread of the Indus Civilisation, there is strong evidence for long-distance exchange of raw materials and finished objects and this process is presumed to involve boats and animal-driven transport, although there is little evidence as to the relative importance of each mode of movement. Strontium isotopic analysis of animal remains from four sites analysed for this study combined with results from nine other sites indicates limited long-distance animal movement between different geological zones within the Indus Civilisation. These findings suggest that individual animals primarily moved short- or medium-distances, though there are several significant exceptions seen in some pigs and cattle found at two large urban sites. We infer that long-distance transport of goods, be it raw materials, finished objects, other goods, or the animals themselves, could have occurred through the use of boats and waterways, by traction animals moving over long distances that did not end up in the archaeological record, and/or by different animals participating in many short to medium-distance movements.

## Introduction

Complex societies provision their populations with the resources necessary for life, and this includes staples, fuel and also items made from rare and more exotic materials. Studies of material culture and material provenance inform us about the sources of raw materials and the locations between which goods were moved. While iconographic depictions of boats, carts and animal and human traction can provide insight into the available mechanisms for moving those goods, it is not always simple to reconstruct the organisation and relative importance of different modes of transport.

Archaeologists have long debated the significance of exchange and trade^1–7^. South Asia’s Indus Civilisation is an interesting case in point, as the settlements of its urban phase (2600–1900 BC) were distributed over an extensive area of Pakistan and northwest India, and there is abundant evidence for the movement of raw materials and finished products made from different types of metal and stone, as well as stoneware bangles and large storage vessels over short, medium and long distances^8–11^. Compositional analyses have suggested that these raw materials and finished objects were widely distributed (and redistributed) across the Indus Civilisation, being brought from sources as far afield as northern Afghanistan, Khyber Pakhtunkhwa, the Himalayas, Maharashtra, Rajasthan, and Baluchistan^11^. Archaeometric studies have shown that manufactured items such as stoneware bangles and carnelian beads were moved within the Indus Civilisation^9,12,13^. Indus-type artefacts were exported overland across complex highland terrain as far as Badakhshan in Afghanistan and the Kopet Dagh piedmont in Turkmenistan. Material was also moved by water throughout the Persian Gulf, and to cities in southwest Iran and Mesopotamia^9,14–18^.

The different scales of movement that took place within the Indus Civilisation largely remain unspecified, and discussion of the logistics of how goods were transported over these distances has largely been speculative. Across the expanse of the Indus Civilisation, short distances were feasibly those traversable on foot or hoof in one day (up to 25 km), medium distances would require several days of travel, potentially within one geographical region (up to 100–150 km), while long distance movement would involve travel over hundreds of kilometres and potentially weeks of travel time, and include movement between different geographical regions. To date, the assumption has been that raw materials and goods were moved over medium and long distances across the Indus Civilisation through a combination of riverine and animal transportation^9,19–21^. Indus boats are attested by depictions on Indus seals^9,20^, while animal transport is indicated by a relative abundance of clay cart models and animal figurines, zooarchaeological evidence for animals being used for traction, and cart tracks in the streets of Harappa^9,19–22^.

The traditional view is that the Indus Civilisation was riverine, with settlement distribution biased towards locations close to perennial water sources^20,21^. While there were clearly settlements on rivers and smaller water courses, there is also abundant evidence that settlements were situated in a variety of other contexts not close to obvious perennial or flowing water sources, and this is particularly the case for settlements distributed across northwest India^21,23–27^. Furthermore, a significant proportion of the ancient hydrology that has been reconstructed across the Indus River Basin was likely composed of ephemeral water courses for much of the Holocene^26,28–32^. It is still unclear how many of these water courses would have been navigable, even to small boats with shallow draft. Given the degree of variability in settlement location, and the likelihood that not all settlements were interconnected via the hydrological system, animal transport must have played an important role in some Indus long-distance exchange logistics, whether for complete or partial journeys.

Indus Civilisation boat transport is likely to have been relatively simple where navigable water courses were present, relying on wind, current, animal and human power for motion. Some of these watercraft may have been connected to specific settlements, but their crews must also have interacted with other actors, as the settlements in interstitial areas away from navigable rivers are likely to have only been accessible using animal driven and/or human transport. Kenoyer^19^ has suggested that appropriately loaded animals of various sizes may have been used to move goods over shorter distances, but noted that carts would have been more efficient, estimating that individual Indus carts could hold 1870 kg of goods. He has suggested that carts were ideal for transporting bulk goods over short distances across the plains, but argued that the lack of roads and bridges would have made long distance transport of goods via carts difficult^19^. Thus far it has been challenging to test this hypothesis. It is likely that the logistics via which the different types of transportation operated were dependent upon the types of individuals and economic transactions involved.

The nature of the political economy of the Indus Civilisation is much discussed, and the role of exchange and trade in empowering and enriching individuals or groups within cities and other settlements is debated^33–35^. Heather Miller^20^ has highlighted the clear evidence for the operation of way-stations and road markers as critical parts of the overland transport system under the Mughal Empire, but no such structures have yet been identified in the context of the Indus Civilisation. Although there are a range of inscribed materials that are indicative of administrative activities^35^, the Indus script remains untranslated, and there is an absence of records that provide explicit insight into the administration of exchange processes. There is evidence for the use of a standardised weight system^36,37^, but these weights were typically in small units, and were not likely to have been used for bulk goods^38^. It is possible that the sourcing and distribution of raw materials and finished products within the Indus economy was part of an elite driven and controlled system that involved merchants and places of exchange. In a formalised system with merchants potentially acting as independent agents, individual teams of transportation animals may have been used to move bulk goods long distances, or individual loads may have been moved by many teams over shorter distances. Both options would have required significant logistical planning. It is also possible that a more substantive system of sourcing and distributing raw materials and finished products may have been operating, where the distribution of goods and the movement patterns of transport teams may have been governed by redistribution relationships and reciprocity. Such a system may show patterns more in keeping with distance-decay or down-the-line exchange models, where the distribution of materials declines with distance from the source^4,39,40^. Ideally, analysis of archaeological material remains provides the means of ascertaining the nature of ancient exchanges systems, though the interpretation of data is not always straightforward.

Strontium isotopic analysis allows us to examine whether animals travelled long-distances, as it enables us to identify animals born in a different geological zone to the one in which they were deposited archaeologically. In the context of the Indus Civilisation, establishing whether animals moved beyond individual geological ‘isozones’ has implications for understanding short, medium and long-distance interaction in the Indus River Basin. In particular, it has the potential to provide evidence in support of the hypothesis that carts drawn using animal traction were used to transport goods long distances. Here, we analyse 94 samples from 39 animals from four Indus settlements in northwest India. We compare these data with previously published animal and human enamel strontium isotope data from nine settlements in other areas within the Indus Civilisation. By considering the dynamics of animal mobility, we explore the degree to which animal movement occurred at long-range scales between settlements within different parts of the Indus Civilisation, and make inferences about the degree to which it operated at local to medium scales between villages, and between villages and neighbouring cities.

## Approaching animal mobility in the Indus Civilisation

### The Indus Civilisation and evidence for animal use

**Figure 1:**
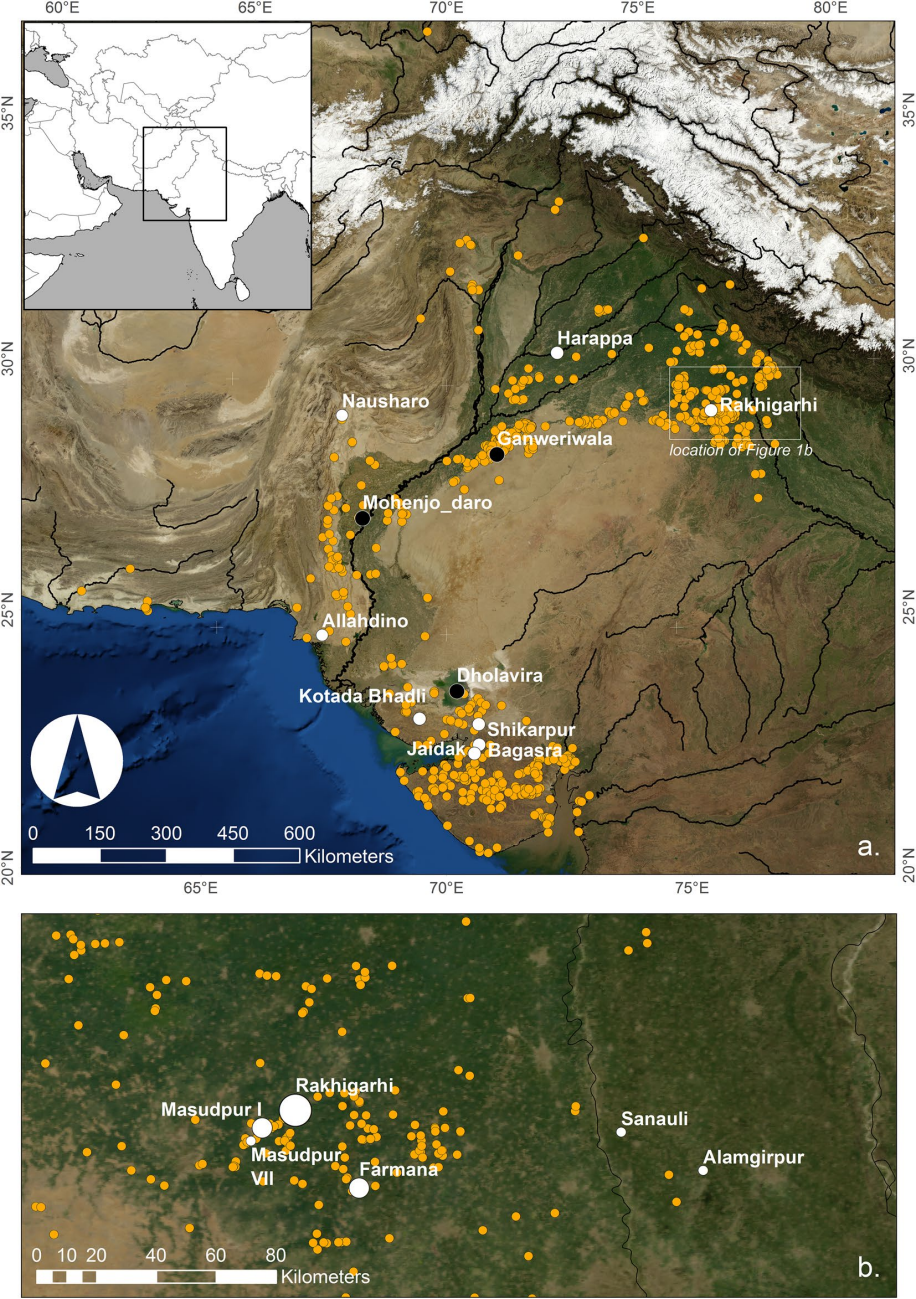
Maps of sites mentioned in the text: (**a**) Map of the Indus Civilization with known sites shown by orange circles, city sites by black circles and sites with animal strontium isotope data by white circles; (**b**) Map of the study region, with white circles indicating the sites in Haryana analysed as part of this study, where the size of the circle relates to the site size in hectares [images generated using ArcGIS and NASA Blue Marble imagery].

The Indus Civilisation is characterised by the appearance of the first cities in South Asia, but was also marked by a preponderance of rural settlements distributed across a large area of modern Pakistan and India (Fig. 1). Cattle have long been regarded as being tremendously important to the Indus subsistence economy^41,42^, and largely it has been assumed that cattle were the primary beasts of burden^22,43,44^. Indus zooarchaeological assemblages typically have a high proportion of cattle (largely *Bos indicus*) and/or water buffalo (*Bubalus bubalis*), typically 50 to 60%, but also occasionally up to 80 to 90%, and a smaller proportion of sheep/goat (*Ovis aries* and *Capra hircus*), with some pig (*Sus domesticus*), wild terrestrial and aquatic bones also being found^22,45,46^. Laura Miller^22,44^ argued that large bovids, including both zebu and water buffalo, were the animals most suited to heavy traction in both agricultural production and the transport of the resultant products, as well as of raw materials and craft items. She also observed that 90% of bovine animals survived into reproductive adulthood (3–3.5 years), at which point they were exploited for secondary products potential—females as dairy producers and males/castrates as traction animals [22:484, 625–628]. Cattle bones reveal evidence for pathological conditions associated with the physical stress associated with traction and heavy duty labour. The widening of the distal end of the metapodia, pedosis, and exostosis of the proximal ends of the third phalanges is seen at a number of sites, including Harappa^22,44^, Farmana^47^, Kanmer^48^, Surkotada^49^, Jaidak^50^, Shikarpur, Kuntasi and Karanpura in Rajasthan^46^.

### Strontium isotope ratios and movement

Strontium isotopic analysis can be used to identify animals that moved between geological zones^51,52^. This technique is based on the principle that strontium isotope ratios (^87/86^Sr) in tooth enamel reflect the geology from which an individual’s food was sourced^53–57^. The level of strontium isotopic variation in any region is dependent on a number of factors, including underlying geology, weathering rates, soil composition and alluvial overburden. The level of geological variation in any region is variable, but on large alluvial plains such as those in Haryana and Indian and Pakistani Punjab, geological variation is more likely over long-distance scales than it is at local and potentially medium-distance scales.

Non-local individuals are identified as those whose tooth enamel strontium isotope ratios are not reflective of the local environment^52^. The identification of such a mis-match is not always simple, particularly in environments where the local isotopic signal is not well-constrained. In practice, a combination of techniques is often used, including: comparison to previously analysed individuals from the region (human or animal); comparison to geological maps; comparison with modern strontium isotope ratios of the target region determined by analyses of water, sediment, plant, snails and/or small mammal samples; and statistical analysis of the dataset^58–60^. All of these methods have flaws, particularly with regards to whether or not modern data truly reflects the past and/or ‘missing’ data, and it is therefore sensible to pursue a multi-strand strategy.

### Geological and strontium isotopic variation in the Indus region

**Figure 2:**
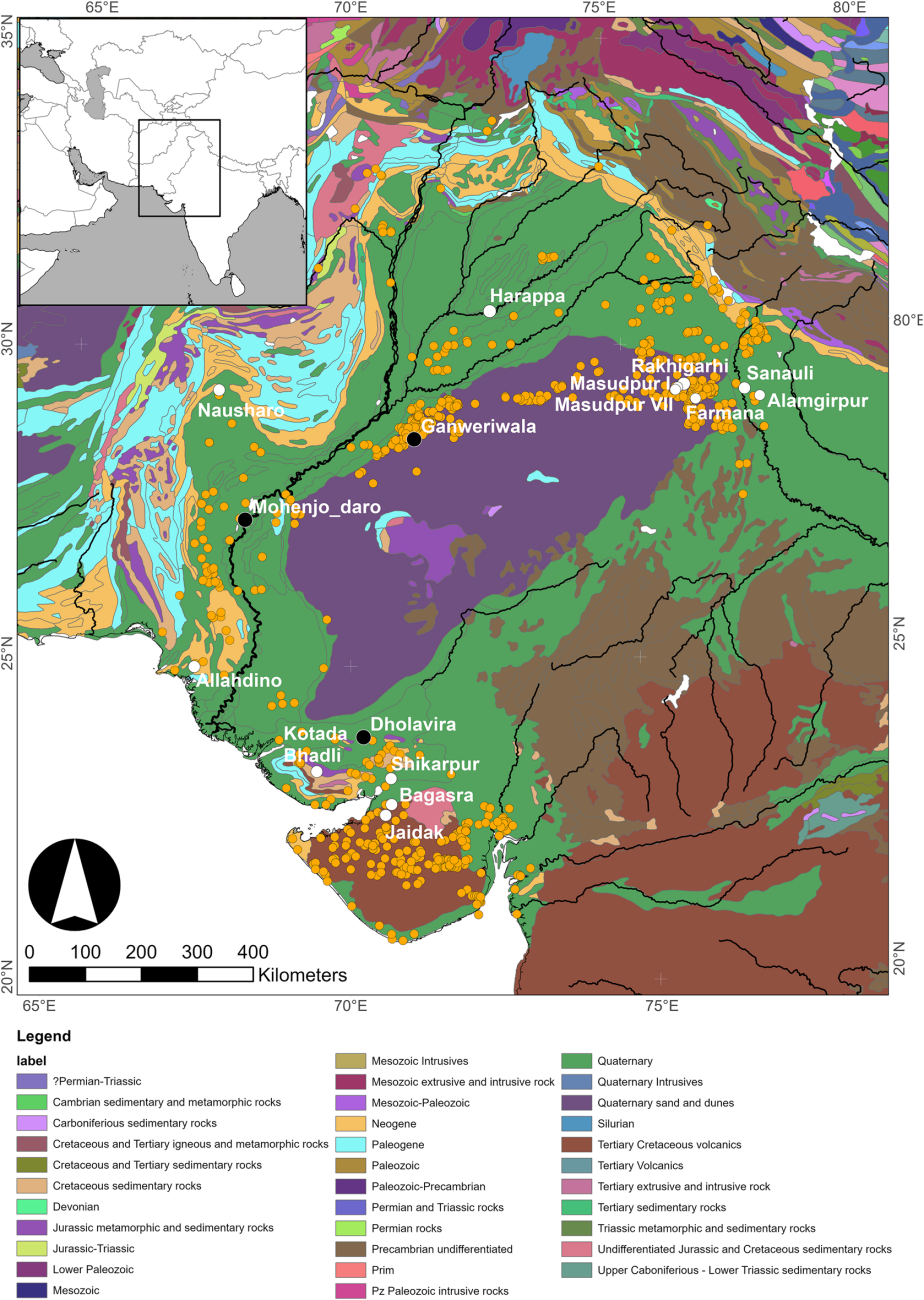
Geological map of the Indus River Basin, with known sites shown by orange circles, city sites by black circles and sites with animal strontium isotope data by white circles [images generated using ArcGIS and USGS World Geological Maps Data].

The Indus River Basin and surrounding regions are characterised by a range of different geological units. Much of the lower basin is comprised of Quaternary alluvial and sand/dune deposits (Fig. 2) and the archaeological sites included in this study are all located on the alluvial plains, where alluvium has been deposited by the Indus and the other rivers of Punjab, and is typically hundreds of metres deep^61–64^. Alluvium in Hissar District (India), where four of the study sites are located, varies from 228 to 310 m in depth overlying pre-Cambrian granite and mica schist bed-rock^61^*.* The north of the Indus River Basin is bordered by mountain chains, including the Karakorum, the Salt Range and the Himalayas, which include Paleozoic, Mesozoic, Palaeogene, and Neogene geology. The eastern edge of the basin is formed by the Thar Desert, which consists of sand and dunes formed by Quaternary sediments. Gujarat, to the south-east of the basin, is dominated by the Deccan Traps, which is a large volcanic feature that formed in the Cretaceous period. Finally, the Western Fold Belt lies to the west of the area and includes two major mountain chains, the Kirthar and Suleiman Mountains, which are largely Paleogene and Neogene in age. To the east of the Thar Desert and bordering on the alluvial plains of Haryana is the Proterozoic Aravalli Range.

River water and sediment samples show strontium isotopic variation across these zones and suggest that there are three inputs: the high Himalayan crystallines, the Kohistan Arc, and the Western Fold belt, which lead to strontium isotopic variation within the Indus River Basin and the neighbouring regions^65–67^. Variation is also seen in biologically available strontium isotope ratios of soils, modern dung and archaeological samples of animals and humans which fits broadly with the geological patterns (summarised in Table 1 and references therein).

**Table 1:** Summary of strontium baseline data for the Indus civilisation area.

Site name	State	Sample type	Sample n	Range	Estimated 'local range' (n)*	References
Rakhigarhi	Haryana	*Sus* tooth enamel	8	0.71473 to 0.71905	0.71558 to 0.71587 (5)	Valentine et al.^77^
Farmana	Haryana	Soil leachate	6	0.71555 to 0.71596	0.71555 to 0.71566 (5)	Valentine et al.^77^
Farmana	Haryana	Human tooth enamel	37	0.71531 to 0.72040	0.71551 to 0.71602 (29)	Valentine et al.^77^
Sanauli	Uttar Pradesh	Soil leachate	6	0.71754 to 0.71969	0.71754 to 0.71969 (6)	Valentine^70^
Sanauli	Uttar Pradesh	Human tooth enamel	66	0.71256 to 0.72716	0.71695 to 0.72218 (64)	Valentine^70^
Harappa	Pakistani Punjab	Human tooth enamel	88	0.71113 to 0.72802	0.71113 to 0.72190 (85)**	Kenoyer et al.^74^; Valentine et al.^77^
Harappa	Pakistani Punjab	Faunal tooth enamel	22	0.71569 to 0.72112	0.7167 to 0.71913 (18)	Kenoyer et al.^74^; Valentine et al.^77^
Mehrgarh	Balochistan	*Canis* tooth enamel	2	0.70804 to 0.70817	0.70804 to 0.70817 (2)	Valentine^70^
Mehrgarh	Balochistan	Soil leachate	4	0.70793 to 0.70813	0.70793 to 0.70813 (4)	Valentine^70^
Nausharo	Balochistan	Faunal tooth enamel	6	0.70812 to 0.70828	0.70812 to 0.70828 (6)	Valentine^70^
Allahdino	Sindh	Faunal tooth enamel	5	0.70871 to 0.71090	0.70871 to 0.71090 (5)	Valentine^70^
Balakot	Sindh	Soil leachate	6	0.70870 to 0.70897	0.70884 to 0.70897 (5)	Valentine^70^
Bagasra	Gujarat	Faunal tooth enamel	262	0.7087 to 0.7099	0.7090 to 0.7097 (242)	Chase et al.^81^; Chase et al.^76^
Jaidak	Gujarat	Faunal tooth enamel	68	0.7089 to 0.7102	0.7092 to 0.7098 (64)	Chase et al.^76^
Shikarpur	Gujarat	Faunal tooth enamel	137	0.7091 to 0.7106	0.7093 to 0.7096 (125)	Chase et al.^76^
Kotada Bhadli	Gujarat	Faunal tooth enamel	18	0.7093 to 0.7104	0.7094 to 0.7104 (16)	Chakroborty et al.^75^
Multiple	Gujarat	Dung	125	0.7081 to 0.7104	n/a (basemap covers state)	Chase et al.^78^

*Local ranges estimated from the interquartile range (IQR). **In this instance the human strontium isotopic data are bimodal (and the analysed individuals likely contain a high proportion of migrants—see text for further discussion), so a local range derived from the IQR is likely inappropriate; the faunal data probably represent a more reliable local range.

## Materials and methods

We collected and analysed 15 soil samples and 94 samples of enamel from 39 animal teeth following standard methods. The Supplementary Information (SI) contains full details of sample collection, preparation and analysis of strontium concentrations and ^87/86^Sr ratios (hereafter strontium isotope ratios).

Soil samples were collected from locations distributed along a cross-shape across Haryana to test for systematic variation based upon the spread of the aeolian sands from the southwest and the sediment deposited by rivers from the north and northeast (see SI 1, Fig. SI 1, Table SI 1). Animal teeth were chosen from four archaeological sites where both cattle/water buffalo and sheep/goat were attested (Fig. 1; SI 2, Table SI 2). Samples of cattle, water buffalo, sheep, goat, pig and boar were taken where available, however sample size was limited by extremely poor preservation of the remains^68^. Two of these sites, Masudpur I and Masudpur VII, are in close proximity in central Haryana, Farmana is *c*.40 km to their southeast, but also on the plains of Haryana. The fourth site, Alamgirpur, is located *c*.100 km further to the east of Farmana, on the other side of the Yamuna River in Uttar Pradesh, adjacent to the floodplain of the Hindon River, which flows through the Ganges/Yamuna doab. For the 28 hypsodont teeth (i.e. cattle, water buffalo, sheep/goat) three samples were taken from each tooth, from the top, middle and bottom of the crown, while bulk samples were taken from the 11 pig/boar and wild animal teeth (SI 3).

Figures, data summaries were generated, and statistical analyses (SI 4, SI 5, SI 6) were performed using Rstudio version 2022.12.0 + 353^69^. Kruskal Wallis tests with post-hoc Wilcoxon rank sum tests were used since the data were non-parametric. Outliers identified statistically are defined as samples that lie more than 1.5 times the interquartile range from quartile 1 or 3^59^.

## Results

### Animal tooth enamel strontium concentration values

The enamel samples show a wide range of strontium concentrations (239 to 2418 ppm, n = 94; Fig. SI 2, SI 3), which is higher than has been measured elsewhere (e.g. 20–417 ppm in human teeth from archaeological sites on the British Isles)^58^, but consistent with concentrations in previous studies of tooth enamel from sites in India^70^. Herbivore faunal tissues tend to have higher strontium concentrations than omnivores and carnivores because plants are strontium rich compared to meat^71–73^. There is a clear relationship between strontium concentration, region and site: at Alamgirpur, the concentration values fall between 239 and 488 ppm, while at Masudpur I, Masudpur VII and Farmana the concentrations are much higher (494 to 2418 ppm, collectively). This geographical pattern is supported further by previous strontium concentration analyses of animal and human teeth from Harappa, Rakhigarhi, Farmana and Sanauli^70,74^ (Table SI 3; Fig. SI 2). We therefore suggest that these animal tooth enamel concentration values are generally reflective of particular geological zones rather than contamination.

### Comparison between sites and identifying strontium isozones within the Indus Civilisation

**Figure 3:**
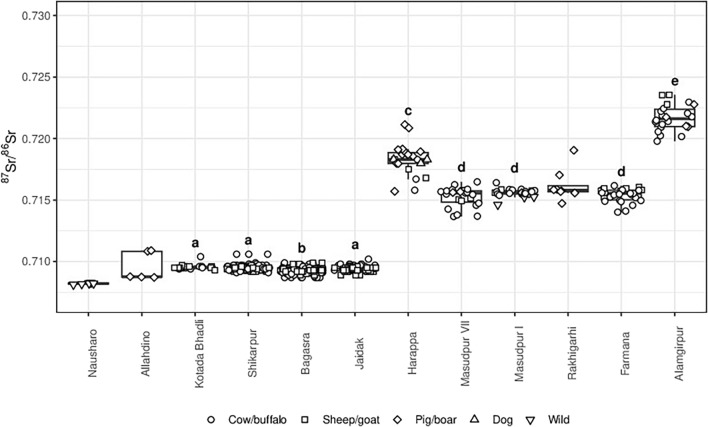
Boxplot of animal enamel strontium isotope results from the Indus Civilisation summarised by site, specifically previously published material from Nausharo (n = 6), Allahdino (n = 5), Kotada Bhadli (n = 18), Shikarpur (n = 137), Bagasra (n-262), Jaidak (n = 68), Harappa (n = 22), and Rakhigarhi (n = 8), and material for this study from Masudpur VII (n = 23), Masudpur I (n = 23), Farmana (n = 26) and Alamgirpur (n = 22). Letters a-e refer to the results of post-hoc statistical analyses, which exclude the three sites with n < 18 (Nausharo, Allahdino, Rakhigarhi; see text for details). Outliers are those which are more than 1.5IQR beyond Q1 or Q3: three pigs (HS2, HS4, HS5) and one bovid (F3926) from Harappa, one pig (RS5) from Rakhigarhi, see S1 for more information [image generated using R].

The strontium isotope ratios of animal tooth enamel show a clear patterning by site and geography (Fig. 3; Table 2). Animals from the sites from the plains of Haryana in northwest India presented here (Farmana, Masudpur I, Masudpur VII) are similar to each other and also to those from Rakhigarhi^70^ in terms of strontium isotope ratios. These animals are distinct from those from the site of Harappa, situated in Pakistani Punjab, in terms of strontium isotope ratios^70^. Animals from the sites in Sindh in Pakistan (Nausharo, Allahdino)^70^ and Gujarat in India (Kotada Bhadli, Shikarpur, Bagasra, Jaidak)^75,76^ are distinct and also cluster together. The animals from Alamgipur presented here are clearly different to those from all of the other sites, with higher strontium isotope ratios. It is notable that the animals from the two city sites of Rakhigarhi and Harappa show a wider range overall. Statistical testing supports this clustering based on site location (Kruskal–Wallis test of effect of site: H(357.13) = 124, p < 0.001, sites with n < 18 excluded (Rakhigarhi, Nausharo, Allahdino); post-hoc Wilcoxon rank sum tests indicate that the sites can be categorised into five groups denoted by letters a-e in Fig. 3).

**Table 2:** Summary of animal tooth enamel strontium isotope values from the Indus, by site (all serial sub-samples from individual teeth included).

Site name	Sample n	Isozone code	Mean	Median	Standard Deviation	IQR	Minimum	Maximum	Range	References
Nausharo	6		0.7082	0.70822	0.00006	0.00007	0.70812	0.70828	0.00016	Valentine^70^
Allahdino	5		0.7096	0.70877	0.00116	0.00209	0.70871	0.71090	0.00218	Valentine^70^
Kotada Bhadli	18	a	0.7096	0.70950	0.00023	0.00010	0.70930	0.71040	0.00110	Chakroborty et al.^75^
Shikarpur	137	a	0.7095	0.70950	0.00022	0.00010	0.70910	0.71060	0.00150	Chase et al.^76^
Bagasra	262	b	0.7093	0.70930	0.00018	0.00020	0.70870	0.70990	0.00120	Chase et al.^76^
Jaidak	68	a	0.7094	0.70950	0.00022	0.00020	0.70890	0.71020	0.00130	Chase et al.^76^
Harappa	22	c	0.7183	0.71830	0.00131	0.00088	0.71571	0.72114	0.00543	Kenoyer et al.^74^
Masudpur VII	23	d	0.7153	0.71557	0.00079	0.00092	0.71366	0.71648	0.00282	This study
Masudpur I	23	d	0.7156	0.71560	0.00033	0.00027	0.71462	0.71641	0.00180	This study
Rakhigarhi	8		0.7162	0.71580	0.00131	0.00049	0.71473	0.71905	0.00432	Valentine^70^
Farmana	26	d	0.7154	0.71554	0.00056	0.00079	0.71402	0.71617	0.00215	This study
Alamgirpur	22	e	0.7217	0.72161	0.00105	0.00141	0.71978	0.72354	0.00376	This study

The isozone code is derived from the statistical analyses (see text for details).

Given the groupings identified by the post-hoc Wilcoxon rank sum tests we can infer that there are at least four clear geographic strontium isozones that can be differentiated in the Indus River Basin and neighbouring areas based on the available samples – ‘Gujarat/Baluchistan’ (groups ‘a’ and ‘b’—Kotada Bhadli, Shikarpur, Bagasra and Jaidak; also Nausharo, Allahdino), ‘western Punjab’ (group ‘c’—Harappa), ‘Haryana’ (group ‘d’—Masudpur VII, Masudpur I, Farmana, and also Rakhigarhi which is excluded from the post-hoc tests due to sample size) and the ‘Ganges-Yamuna doab’ (group ‘e’—Alamgirpur). The samples from Nausharo in Baluchistan and Allahdino in Sindh may in fact represent a fifth strontium isozone, though it is one that has similar strontium isotope ratios to Gujarat, which may be a result of similar parent rocks in each region (Fig. 2); the sample sizes are too small to be included in the statistical analyses (N = 6 and 5, respectively). Here the Gujarat and Baluchistan samples (a & b) have been grouped as belonging to one isozone.

In most cases, the enamel strontium isotope ratios are consistent with those of the relevant soil leachate and dung data from this study and previously published samples of dung, shell and soil^70,77,78^, supporting the identification of different isozones (Fig. 3). Soils from Baluchistan have similar values to animal enamel data from Nausharo; soils from Sindh have similar values to animal enamel data from Allahdino; dung and shell from Gujarat have similar values to animal enamel data from Kotada Bhadli, Shikarpur, Bagasra and Jaidak; soils from Haryana have similar values to animal enamel data from Masudpur VII, Masudpur I, Farmana and Rakhigarhi. It is notable, however, that while the tooth enamel results from Alamgirpur are very distinct from the samples from all other sites, they are also different to the samples from Sanauli, which is similarly situated in western Uttar Pradesh (Fig.SI 4). This difference may be due to the fact that each of these sites is located within the watershed of a different river, but it cannot otherwise be explained at present.

### Intra-tooth isotopic variation

**Figure 4:**
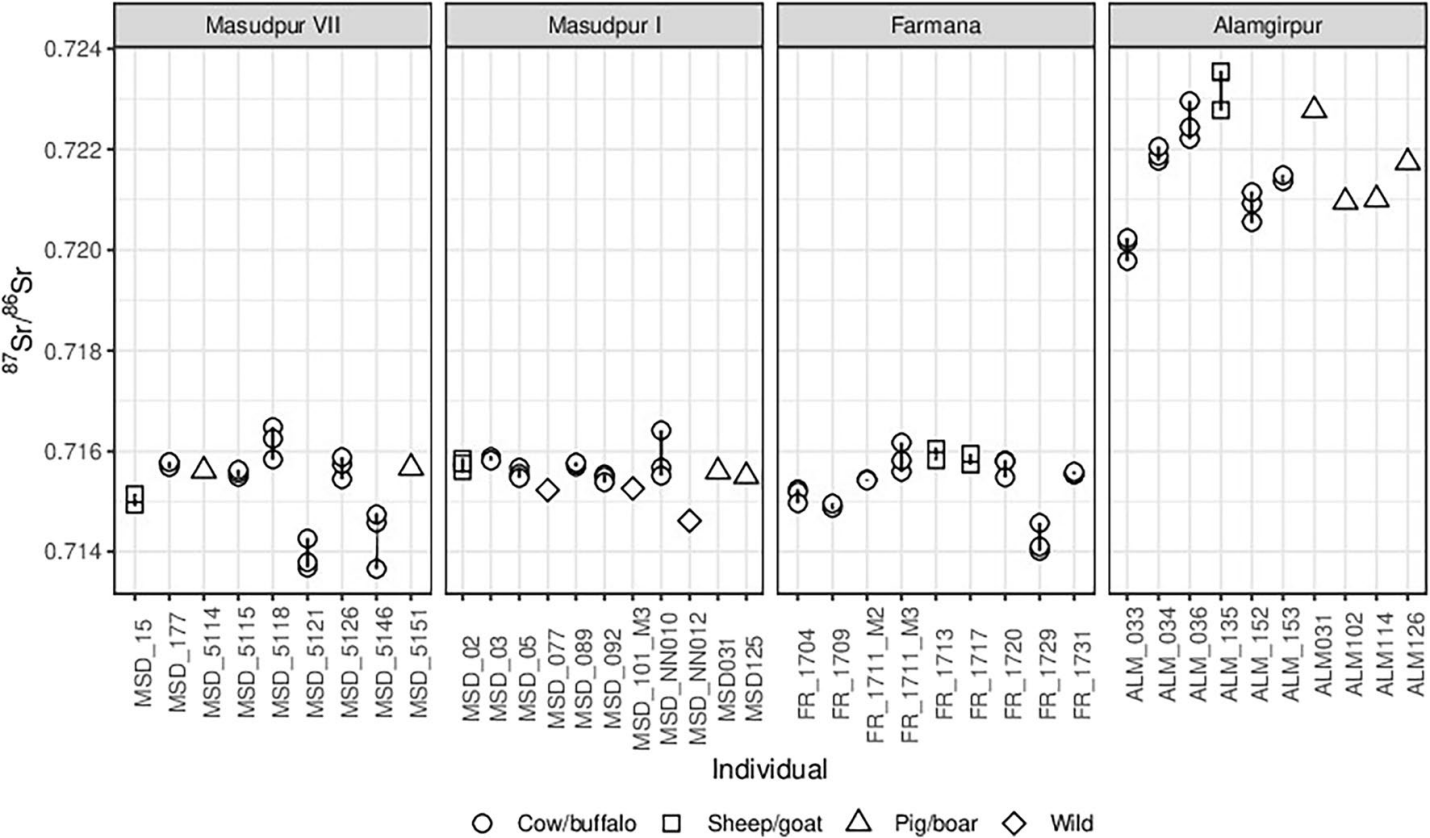
Enamel strontium isotope results from the animal teeth analysed in this study, plotted by site, showing the change within individual teeth [image generated using R].

There is little isotopic variation seen in sub-sampled animal teeth (Fig. 4; Table 3, Table SI 2); for the 28 teeth where we have multiple sub-samples, the mean of the strontium isotopic variation within a tooth is 0.00036 (minimum range = 0.00005, maximum range = 0.00108). The maximum intra-tooth change is significantly less than the total variation seen in the strontium isotope ratios from across the Indus River Basin. The biggest change within an individual tooth is seen in the bovid MSD5146 (from Masudpur VII, range = 0.00108), which we note also had the largest range in carbon isotope values in our previous research, although they still consumed predominantly C_4_ food throughout the time of tooth formation (δ^13^C range = 3.4‰)^68^. The next largest change is seen in the bovid MSDNN010 (Masudpur I, range = 0.00090, identified as an outlier at the site level), although this individual had a more typical range in carbon isotope values (1‰). In total there are nine teeth with intra-individual variation more than 0.0005 their strontium isotope ratios (Table 3), but only three of which appear to deviate from the typical values of fauna at the site level (FR1729, MSDNN010, MSD5121), along with one antelope (MSDNN012) where the tooth was analysed in bulk (Fig. 4). Thus, with a small number of exceptions, these data indicate limited movement across different geological zones during the period of tooth formation for each of the individuals analysed.

**Table 3:** Strontium isotope variation in animal tooth enamel sub-samples from this study by individual.

Individual	Species	Site	n	Minimum	Maximum	Range
ALM_033	Bos indicus	Alamgirpur	3	0.71978	0.72023	0.00045
ALM_034	Bos indicus	Alamgirpur	3	0.72177	0.72205	0.00028
ALM_036	Bos indicus	Alamgirpur	3	0.72221	0.72296	0.00075
ALM_135	Ovis aries	Alamgirpur	3	0.72278	0.72355	0.00077
ALM_152	Bos indicus	Alamgirpur	3	0.72055	0.72115	0.00059
ALM_153	Bos indicus	Alamgirpur	3	0.72136	0.72148	0.00012
ALM031	Sus scrofa	Alamgirpur	1	0.72277	0.72277	0
ALM102	Sus domesticus	Alamgirpur	1	0.72096	0.72096	0
ALM114	Sus domesticus	Alamgirpur	1	0.72101	0.72101	0
ALM126	Sus scrofa	Alamgirpur	1	0.72174	0.72174	0
FR_1704	Bos indicus	Farmana	3	0.71497	0.71524	0.00027
FR_1709	Bos indicus	Farmana	3	0.71487	0.71496	0.00009
FR_1711_M2	Bos indicus	Farmana	2	0.71542	0.71544	0.00002
FR_1711_M3	Bos indicus	Farmana	3	0.71559	0.71617	0.00058
FR_1713	Ovis aries	Farmana	3	0.71582	0.71604	0.00022
FR_1717	Capra/Ovis	Farmana	3	0.71574	0.71594	0.00021
FR_1720	Bos indicus	Farmana	3	0.71548	0.71582	0.00033
FR_1729	Bos indicus	Farmana	3	0.71402	0.71457	0.00055
FR_1731	Bos indicus	Farmana	3	0.71554	0.71559	0.00005
MSD_02	Capra/Ovis	Masudpur I	3	0.71560	0.71585	0.00025
MSD_03	Bos indicus	Masudpur I	3	0.71581	0.71588	0.00007
MSD_05	Bos indicus	Masudpur I	3	0.71547	0.71567	0.00020
MSD_077	B.tragocamelus	Masudpur I	1	0.71523	0.71523	0
MSD_089	Bos indicus	Masudpur I	3	0.71570	0.71577	0.00007
MSD_092	Bos indicus	Masudpur I	3	0.71538	0.71554	0.00015
MSD_101_M3	A.cervicapra	Masudpur I	1	0.71526	0.71526	0
MSD_15	Capra hircus	Masudpur VII	3	0.71494	0.71515	0.00021
MSD_177	Bubalus bubalis	Masudpur VII	3	0.71569	0.71578	0.00010
MSD_5114	Sus scrofa	Masudpur VII	1	0.71561	0.71561	0
MSD_5115	Bos indicus	Masudpur VII	3	0.71549	0.71563	0.00014
MSD_5118	Bos indicus	Masudpur VII	3	0.71584	0.71648	0.00064
MSD_5121	Bos indicus	Masudpur VII	3	0.71369	0.71426	0.00058
MSD_5126	Bos indicus	Masudpur VII	3	0.71545	0.71588	0.00043
MSD_5146	Bos indicus	Masudpur VII	3	0.71366	0.71475	0.00108
MSD_5151	Sus scrofa	Masudpur VII	1	0.71567	0.71567	0
MSD_NN010	Bos indicus	Masudpur I	3	0.71551	0.71641	0.00090
MSD_NN012	A.cervicapra	Masudpur I	1	0.71462	0.71462	0
MSD031	Sus scrofa	Masudpur I	1	0.71560	0.71560	0
MSD125	Sus domesticus	Masudpur I	1	0.71550	0.71550	0

## Discussion

### Indus isozones

The animal tooth enamel strontium isotope ratios presented here and in the published literature indicate that there are distinct geographical ‘isozones’ within the Indus River Basin and surrounding areas that were occupied by populations of the Indus Civilisation (Fig. 3). The lowest values come from Kotada Bhadli, Shirkarpur, Bagasra and Jaidak (groups ‘a’ and ‘b), which are located on the Deccan traps in Gujarat, and are similar to the limited tooth enamel data from Nausharo and Allahdino, which are situated in the more varied geologies of the Western Fold Belt in Baluchistan and Sindh (‘Gujarat/Baluchistan’ isozone; Fig. 2). While it seems counter-intuitive that such different base geologies would have similar strontium isotopic signatures, these data are broadly consistent with the soil leachate data from these regions (Table 1 and Fig. SI 4), and it is notable that geological formations of similar age are present in both regions (Fig. 2). It may be that further research and analysis will allow for further differentiation, but alternatively it may be that the average strontium isotope ratios of these regions are too similar to be clearly distinguished. Another isozone is represented by the samples analysed from Haryana (group ‘d’ in Fig. 3), and represents at least the Haryana part of the Indus River Basin floodplain (‘Haryana’ isozone). Significantly, the strontium isotopic range of the sites in Haryana is similar to several of the animal tooth samples from Harappa (group ‘c’ in Fig. 3). The clustering of the majority of samples from Harappa indicates that this part of Pakistani Punjab at least (‘western Punjab’ isozone) represents a separate ‘isozone’ to Haryana, potentially demonstrating that sediments deposited in different parts of the Indus River Basin are derived from different Himalayan sources. Alamgirpur sits on an alluvial plain in Uttar Pradesh that is part of a different watershed to the other sites (Fig. 1), and the samples from there appear to represent another isozone (‘Ganges/Yamuna doab’ isozone; Fig. 2, Fig. SI 4).

### Long-distance movement of animals

We can use isotopic variation within the cattle, water buffalo, and sheep/goat teeth that were sub-sampled to consider mobility within the time period of tooth formation for each individual animal. Given the formation times of teeth (1–2 years for hypsodont teeth) and typical animal lifetimes (6–12 years)^79,80^, it is a fair assumption that long-distance movement between different isozones will be reflected in animal tooth enamel strontium isotope ratios. As mentioned above, 9 of the 28 animals that we sub-sampled from the sites in Haryana had strontium isotopic variation greater than 0.0005 within their teeth. Nevertheless, all of the strontium isotope ratios of these individuals more closely match to the strontium isotopic data from their geographic area than they do with the strontium isotopic data from elsewhere in the Indus Civilisation. The implication is that there is likely to have been some movement of animals between areas that were not very isotopically distinct, most likely relatively local movement. This conclusion is echoed in the data from isotopic analyses previously performed at Bagasra and Kotada Bhadli^75,81^. Indeed, the only strong evidence for animal movement between ‘isozones’ is one pig from Rakhgarhi, as well as several pigs and potentially one or two bovids from Harappa (evident in Fig. 3). This pattern indicates that pigs moved between isozones, presumably as part of a living shipped load rather than on their own feet, or as a whole carcass, given that the transport of a butchered pig’s head with teeth is unlikely. The possible movement of meat rather than living animals also raises questions about measures of preservation such as salting or smoking, which would have been needed in an environment where spoilage would be a major issue^82^.

Our results resonate with other work on animal mobility, which has shown that humans transported pigs across long distances in the Neolithic in Britain^82^, between Greece and the Levant in the Bronze Age^83^ and across the Pacific during the Lapita migration^84^. The finding that pigs may have been moved around the Indus River Basin, would also seem to provide further warning against the uncritical use of pigs as a means of determining a ‘local signal’ for strontium isotopic analysis.

In summary, the available strontium isotopic evidence suggests that there was limited animal movement between ‘isozones’, but also indicates that some animals moved over long distances. There is some evidence for the existence of different breeds of cattle and sheep within the Indus Civilisation^22,42^, which implies at least some degree of genetic isolation between groups of animals, presumably based on either geography or husbandry practices. Stable carbon and oxygen isotopic data on tooth enamel for many of the animals analysed in this and other studies shows that water buffalo and cattle consumed diets largely based on C_4_ resources, while sheep and goat consumed varying proportions of C_3_ and C_4_ plants throughout the year^68,75,81^. These dietary differences may reflect different local or regional mobility patterns, with sheep and goats likely to have been more mobile than cattle and water buffalo on a daily and seasonal scale.

### Long-distance movement and the Indus Civilisation

The animal tooth enamel samples that have been analysed to date suggest that there was very limited long-distance movement of bovids within the Indus Civilisation. There is only one bovid from Harappa that appears to have originated in Haryana (Fig. 3). The available data thus suggests that typically cattle were being utilised locally, most probably as beasts of burden in support of local-scale farming and traction, facilitating movement between villages, and/or between villages and the larger settlements in their local area. These animals will also have been used for secondary products and food.

How then do we explain a scenario where it is clear that goods, people and potentially multiple pigs were moving long distances within the Indus Civilisation, but cattle and water buffalo were not? We argue that there are three plausible scenarios to explain these data: (i) either cattle (and water buffalo) were not moved or used for long distance transport at all; or (ii) animal-drawn transport was used over long distances, but cattle haulage teams were changed after short distances, perhaps around 25 km. There is also the possibility that: (iii) cattle that moved long distances were not disposed of in the same way that cattle used for other purposes were, and with one exception these animals have not (or have not yet been) found in the archaeological record. While this latter scenario is entirely possible, it stands in contrast to the identification of several ‘migrant’ pigs. All three of these options suppose the existence of a formalised transport and exchange economy.

If, however, we accept that cattle were not moving far or being used for long distance transport at all or at least very rarely, then we are left in a situation where we must hypothesise about the mechanisms of long-distance movement of materials, goods and some animal species. Possibilities include the use of multiple teams of cattle, other animals, boats and/or human-power to move goods over various distances. Of the other potential beasts of burden, the obvious candidate would be donkeys, however donkeys are only found in very low numbers in Indus zooarchaeological assemblages (and horses not at all)^85^. Donkeys therefore do not seem to be a realistic answer to the problem of Indus overland transport. Boats may provide a partial solution but, as noted above, not all settlements were situated on rivers, and many rivers were not navigable all year round. Movement of goods via human moved hand carts is entirely possible, but would not seem an efficient way to move bulk goods long distances in a context where there is evidence for the use of carts and animal traction. The parsimonious explanation given the current data would seem to be a combination of the above mechanisms, with boats used where possible, and probably being responsible for the longer distance movement along the larger, navigable rivers, including the Indus River itself. Given that navigable rivers did not reach every settlement, however, more local and regional (i.e. small and medium distance) distribution of goods almost certainly utilised animal transport to move commodities between key settlement nodes, and from those nodes to outlying settlements. The use of carts and cattle is clearly attested to in Indus material culture, most notably in terracotta figurines, which include abundant examples of cattle and water buffalo. Given the large strontium ‘isozones’ seen in the Indus Civilisation (Fig. 2), such movement on a scale of tens rather than hundreds of kilometres would not necessarily be apparent in the strontium isotopic analyses of Indus animal assemblages.

The analysis of exchange and trade in the Indus Civilisation is a major topic that warrants significant attention and re-evaluation, and a full assessment is beyond the scope of this paper. Law’s^11^ analysis of raw material distribution has demonstrated that non-perishable materials and products were being moved considerable distances. However, the mechanics of the exchange and trade systems that underpin this distribution remain poorly understood. The analysis of animal mobility patterns is a small but, in some ways, critical component of our ability to characterise broader patterns of movement and exchange. The strontium isotopic data presented here suggests that long range movement of individual animals or teams was either limited or not typical. This evidence thus suggests that if raw and finished materials were moving as part of a formal exchange or trade system operating overland, this system must have involved individual haulage teams moving short distances, which were then being switched for new teams so that the cart and/or goods could continue on. The limited evidence for long-distance movement of transport animals might indicate the lack of a formal exchange or trade system involving merchants who controlled transport. However, in terms of the animals themselves, a formal system involving individual teams moving short distances would not be distinct isotopically from down-the-line or distance-decay models, which also involve multiple short distance movements between settlements. The analysis of bovids for strontium thus narrows the range of possibilities, but does not provide definitive answers. It should be possible to differentiate the various explanations through the quantification of material and spatial analysis of material distribution, but this approach has not yet been attempted systematically in the Indus context, and remains an obvious topic for future analysis. It thus remains unclear whether the exchange system (or systems) that operated within the Indus Civilisation involved merchants controlling multiple animal teams or a much less formalised form of down-the-line exchange.

It is somewhat surprising that there appears to be clear evidence for the movement of pigs, with individuals apparently originating in the ‘Ganges/Yamuna doab’ and ‘Haryana’ isozones being found at Harappa, and one individual originating in the ‘western Punjab’ isozone being found at Rakhigarhi. These cities and isozones are hundreds of kilometres apart, and while it is likely that these pigs were moved while alive, they were presumably not walking the whole way at any great speed. Were they being transported in carts drawn by cattle for at least part of the journey? Were multiple cattle teams potentially involved? The clear evidence for the movement of pigs actually fits with the evidence for the movement of various types of raw materials and finished products, but most likely they would have been a living product, and the cultural practices and economic rationales underpinning the choice to move pigs between regions requires further investigation.

Although this paper has focused on the results of strontium isotopic analysis of animal tooth enamel, it is important to give some consideration to the similarities and differences between the strontium isotopic evidence for animals and that of humans^74,77^. There are notably a higher proportion of individuals identified as non-local/migratory within the available human datasets (Fig. SI 4), and the human samples from Harappa have an extremely wide range of strontium isotope ratios, with almost half of the individuals identified as non-local^74,77^. This high proportion of human migrants likely reflects the fact that Harappa was a large city and it is reasonable to conclude that in-migration was highest at such settlements. Importantly, the presence of non-locals is attested in all three of the cemeteries that have had samples analysed, including at Farmana (located in the ‘Haryana/Punjab’ isozone) and Sanauli (located in the ‘Ganges/Yamuna doab’ isozone), although on a smaller scale (Fig. SI 4)^70,77^. It would be revealing to conduct strontium isotopic analysis on human remains from the cities at Mohenjo-Daro, Rakhigarhi and Dholavira, and the smaller sites of Rupar, Lothal and Kalibangan, which all have human remains and/or burials^86^. The cemeteries at Farmana and Sanauli are smaller than Harappa, but the evidence for non-locals at each indicates that human mobility was not simply restricted to movement between cities, and human movement between rural settlements and cities in other regions was potentially prevalent.

## Conclusion

Strontium isotopic analysis of Indus animals has shown very low levels of long-distance animal movement, which contrasts to the considerable evidence for movement of raw materials and finished products, and humans. The exception appears to be pigs. When animals moved long distances, they appear to have ended up in cities. While it is likely that boats were used to move goods, animals and people, the lack of perennial, navigable rivers reaching many settlements means that some animal transport was essential, particularly for moving commodities over short and medium distances, and especially from key nodes to settlements inaccessible by water. Animal-powered transport thus most likely operated on a more local and regional level. If animal-powered transport was used in long-distance movement overland, it remains unclear as to whether it occurred as part of a formalised system where individuals or teams of cattle hauling carts only travelled short distances and were replaced so that the carts and their loads could keep moving. It is entirely possible that the system was less formalised, and products were moved and then unloaded after short distances, and any movement of goods over longer distances was via a system of down-the-line or distance-decay exchange. There is clearly considerable scope for future research into and theorising about the systems of exchange and movement in the Indus River Basin. Amongst a range of possible approaches, the Indus case is ideal for exploration through network and agent based modelling that consider the distribution of individual products, their quantities and the ways in which these patterns change over time.

### Supplementary Information

Supplementary Information.

## Data Availability

All of the data presented in the paper is made available in the tables that are included in the paper and the supplementary information.
