# Supplementary material for: Animal movement on the hoof and on the cart and its implications for understanding exchange within the Indus Civilisation
Source: Sci Rep. 2024 Jan 2;14:158. doi: 10.1038/s41598-023-50249-3 (PMC10762248; doi:10.1038/s41598-023-50249-3)
Supplement: Supplementary file 1 — Supplementary Information. [file 41598_2023_50249_MOESM1_ESM.pdf]

SUPPLEMENTARY INFORMATION (SI)

SI 1. Soil samples

Samples were taken for strontium isotope ratio baseline analysis at a depth of approximately 30 cm and as far away from arable agricultural activities as reasonably possible. Sampling locations are shown in Fig SI1. The soils were leached by: centrifuging at 250 rpm in ultrapure water for 24 hours, then ultrasonicated for 1 hour then centrifuged for 15 minutes at 2000 rpm, then filtered through 0.45 µm PTFE Filters. The routine ashing [1] of the samples were carried out in quartz crucibles using a muffle furnace, with a starting temperature of 300ºC which was increased by 100ºC each hour to a final temperature of 650ºC. The furnace was held at 650ºC overnight (for at least 12 hours), after which it was allowed to cool before ±20 mg of ashed material was weighed into 7 ml Savillex PFA vials, covered with 4 ml of conc $HF:HNO_3$ (1:4 v/v ratio) and placed on a hot plate set to 140ºC for at least 48 hours to dissolve. The sample vials were then opened, dried down on a hot plate set to 100ºC S and converted to nitrate by addition of a few drops of concentrated aq. HNO_3 followed by drying down again, and the process was repeated a second time. The samples were then ready to be analysed for strontium isotope ratios as described below for the enamel samples.

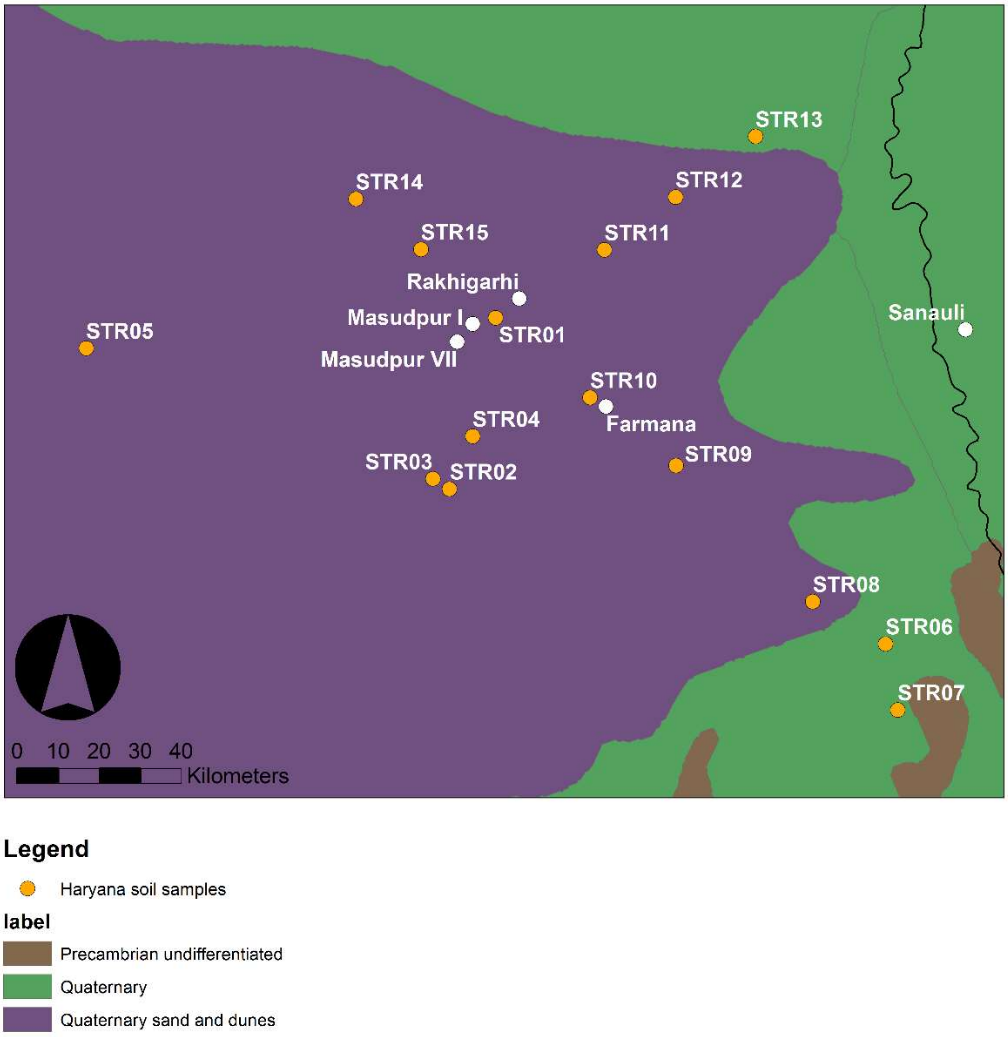

Figure SI1. Sampling locations of soil samples collected from Haryana [images generated using ArcGIS and USGS World Geological Maps Data].

SI 2. Archaeological chronology and site descriptions
A number of different chronological schemes have been used to characterise the Indus Civilisation, but they typically divide the periods into ‘pre-urban’, ‘urban’ and ‘post-urban’ phases, which are also known as ‘Early Harappan’, ‘Mature Harappan’ or ‘Harappa phase’, and ‘Late Harappan’ phases (3200–2600 BC, 2600–1900 BC and 1900–1600 BC, respectively) [2,3,4,5]. In this paper, we make reference to ‘Early Harappan’, ‘Mature Harappan’, and ‘Late Harappan’ phases, and further divide the Mature Harappan (MHar) into sub-phases: i, ii and iii (2600–2450 BC, 2450–2200 BC and 2200–1900 BC, respectively), which are consistent with previous publications of the *TwoRains* project, and comparable to the chronology from Harappa (i.e. Harappa 3a, 3b and 3c) [6,7]. The use of the different numbering is to emphasise the regional difference between these sites and Harappa.

Archaeological sites
<u>Alamgirpur (ALM)</u>
Alamgirpur (Meerut District, Uttar Pradesh) is the easternmost excavated Indus Civilisation site. Although first excavated in the 1950s [8], the samples included here were recovered from excavations undertaken by Banaras Hindu University in 2008.

The sequence at the site includes Mature and Late Harappan, Painted Grey Ware (PGW), Early Historic and Mediaeval material, which has been confirmed by radiocarbon dating [9]. No obvious evidence of non-local material at the site has been published [9]. The archaeobotanical assemblage from the excavations indicates the presence of barley, wheat and rice as well as legumes and oil seeds [9]. Domestic animal remains include cattle, buffalo, sheep, goat, pig and dog with horse present in the PGW period [9].

Teeth from a total of 10 individual animals were sampled for this study, 5 cattle, 1 sheep and 4 pig/boar. The 2 pigs were sampled from PGW contexts, while all of the remaining samples were taken from Mature Harappan iii contexts.

<u>Farmana (FRM)</u>
Farmana (Bhiwani District, Haryana) is a medium-sized settlement extending over an area of up to 18 ha, though the excavated area is much smaller. It was excavated by the RIHN-Deccan College Indus Project in 2006–2007 and 2008–2009. The material assemblage excavated from the site included a range of material that moved some distance, including objects made from steatite, carnelian and a range of other stones not available locally [10].

All but one of the tooth samples analysed in this study was taken from Locality 1 (Central Area), and the other sample was taken from Locality 2 (FR1709). Locality 1 includes Early Harappan, Mature Harappan i, ii and potentially iii deposits [10]. Archaeobotanical remains from Farmana indicate that during the Mature Harappan i phase there was an emphasis on winter crops, wheat and barley, although millets were present [11]. In later phases, millets became more important and a mixed strategy with more equal emphasis on winter and summer crops was followed [11]. The most prevalent animal species are cattle and water buffalo, with five times more cattle than water buffalo, and goats, sheep, pigs and dogs were also present [12]. Compared to other sites in Haryana, Farmana has a large number of wild animals [13].

Teeth from a total of 8 individual animals were sampled, 6 cattle and 2 sheep/goat. Samples were taken from the Mature Harappan ii phase with sample FR1709 being slightly earlier in date.

Masudpur I (MSD I)

Masudpur I (Hisar District, Haryana) is a settlement site in the hinterland of Rakhigarhi. It was excavated by the *Land, Water and Settlement* project in 2009 and revisited in January and February 2018 by the *TwoRains* project. Topographic survey indicates that the site is currently represented by a c. 6 ha mound, interpreted as a large village or small town.

The site contains Mature Harappan ii and iii phase occupation, and has been extensively dated using radiocarbon dating, which suggests that these periods from c. 2400 to 1900 BC [14,15]. The material assemblage excavated from the site included a range of material that moved some distance, including objects made from steatite, carnelian, lapis lazuli and a range of other stones not available locally [14,16,17].

The archaeobotanical assemblage from the 2009 excavation suggests the common use of barley, rice and millets, with wheat being a very minor component [18,19]. The zooarchaeological assemblage included cow, buffalo, goat, sheep and wild animals including elephant, wolf and wild pig, as well as freshwater carp and mussels [12].

Teeth from a total of 8 individuals were sampled, 6 cattle, 1 sheep/goat and two pig/boar, all from Mature Harappan iii contexts.

Masudpur VII (MSD VII)

Masudpur VII (Hisar District, Haryana) is a settlement site in the hinterland of Rakhigarhi, 5km to the southwest of Masudpur I. It was excavated in April-May and December 2009 by the Land, Water and Settlement project. It is estimated to have covered 1 ha. The site contains Early Harappan, Mature Harappan i and iii, and Late Harappan deposits, which has been confirmed by radiocarbon dating [15]. The material assemblage from the site included a range of material that moved some distance, including objects made from steatite, carnelian and a range of other stones not available locally [14,16,17].

The Early Harappan period archaeobotanical assemblage is dominated by millets with some barley and low levels of wheat and rice. During the Mature Harappan period, the proportions of crops changes with a shift away from millet and towards barley and wheat, while during the Late Harappan period rice and barley dominate with some millet [18,19]. The animal species present include cattle, water buffalo, goat and dog plus various wild species including spotted deer, hare and rohu fish [20].

Teeth from a total of 9 individuals were sampled, 1 goat, 1 water buffalo, 5 cattle and 2 pig/boar. The sheep/goat was taken from Mature Harappan iii contexts, while all other samples were taken from Late Harappan contexts.

SI 3. Animal tooth enamel samples and sampling, and isotopic analysis

For the hypsodont teeth (i.e. cattle, water buffalo, sheep and goat) three samples were taken from the top, middle and bottom of the crown, while bulk samples were taken from the pig and boar teeth. All samples were taken using a hand-held drill with a diamond drill attachment. All teeth and drill bits were cleaned in an ultrasonic bath using MilliQ water

several times prior to sampling. Serial samples were taken at perpendicular increments along a single cusp of the tooth, while bulk samples were taken along the length of the surviving crown.

Most of the samples were analysed in the Department of Geological Sciences, University of Cape Town following routine protocols (see for example [21]). Tooth enamel samples for strontium isotope analysis were weighed into closed Teflon beakers and digested for 1 hour at 140°C in 14M (65%) aq. nitric acid (HNO_3) solution. They were then dried down and re-dissolved in 1.5ml 2M aq. nitric acid (HNO_3) solution for strontium separation chemistry following the procedure described in Pin et al. [22]. The separated strontium fraction for each sample was dried down, dissolved in 0.2% aq. nitric acid (HNO_3) solution and diluted to 200 ppb Sr concentrations for analysis on a Nu Instruments Nu Plasma high resolution multi-collector inductively coupled plasma mass spectrometer (HR MC-ICP-MS). Analyses were referenced to bracketing analyses of NIST SRM987 using a $^{87}Sr/^{86}Sr$ normalising value of 0.710255. All strontium isotope data were corrected for isobaric rubidium interference at 87 amu using the measured signal for ^{85}Rb and the natural $^{85}Rb/^{87}Rb$ ratio. Instrumental mass fractionation was corrected using the exponential law, measured $^{86}Sr/^{88}Sr$ ratios and a reference $^{86}Sr/^{88}Sr$ value of 0.1194. Results for repeat analysis of an in-house carbonate reference material processed and measured with the samples from this study ($^{87}Sr/^{86}Sr$ 0.708915; 2-sigma 0.00003; n=14) are in agreement with long-term results for this in-house reference material ($^{87}Sr/^{86}Sr$ 0.708911; 2 sigma 0.000040; n=414). Total procedural blanks measured during the analysis of these samples were typical for this facility at < 250 pg Sr and therefore negligible.

The remaining samples (n=23, indicated by a BCL code and an asterisk in the sample code in Table SI 2) were analysed at the University of Florida, in the Department of Anthropology's Bone Chemistry Lab, and the Department of Geological Sciences ICP-MS lab following the sample preparation process outlined in Valentine et al. [23] and Valentine et al. [24]. Methods are broadly similar to those outlined above, but these samples were run on a Nu Plasma -1 MC-ICP-MS instrument with all data from this instrument, and the TIMS data reported in Valentine [25] and Valentine et al. [24] relative to a NIST SRM987 value of 0.71024 (2 sigma 0.00003).

In order to be able to directly compare the data from these two sets of analyses, the data from the University of Florida has been adjusted (+0.000015) to account for the difference in the value of the reference material. This adjustment has also been applied to data in Valentine [25] and Valentine et al. [24]. Such a comparison is only possible because the measured value of the NIST standard SRM987 is known for both laboratories. We note that the magnitude of the adjustment is very small compared to the range of data. No reference values were quoted in Kenoyer et al. and here we use the published values without adjustment.

References

[1] Scott, M., Le Roux, P., Sealy, J., and Pickering, R. 2020. Lead and strontium isotopes as palaeodietary indicators in the Western Cape of South Africa. South African Journal of Science 116 (5/6): 6700.

[2] Mughal, M.R. 1970. The Early Harappan Period in the Greater Indus Valley and Northern Balochistan (ca. 3000-2400 BC) Ph.D. Thesis. Department of Anthropology, University of Pennsylvania, Philadelphia.

[3] Kenoyer J.M. 1998. *Ancient Cities of the Indus Valley Civilization* Oxford: Oxford University Press.

[4] Possehl, G.L. 2002. *The Indus Civilization: A Contemporary Perspective* Walnut Creek, AltaMira Press.

[5] Wright, R.P. 2010. *The Ancient Indus Urbanism, Economy, and Society* Cambridge: Cambridge University Press.

[6] Kenoyer, J.M. 1991. Urban Process in the Indus Tradition: A preliminary model from Harappa. In *Harappa Excavations 1986-1990: A multidisciplinary approach to Third Millennium urbanism*, edited by R. H. Meadow. Madison, WI: Prehistory Press: 29-60.

[7] Kenoyer, J.M. 2008. The Origin and Character of Indus Urbanism: New Perspectives and Challenges. In Ancient City: New Perspectives on Urbanism in the Old and New World. Edited by J. Marcus and J.A. Sabloff, Sante Fe, SAR Press/National Academy of Sciences: 183-208.

[8] Sharma, Y.D. 1989. Alamgirpur, in *An Encyclopaedia of Indian Archaeology* Vol. II (A. Ghosh Ed.), pp. 11-14. New Delhi: Munshiram Manoharlal.

[9] Singh, R.N., Petrie, C.A., Joglekar, P.P., Neogi, S., Lancelotti, C., Pandey, A.K. and Pathak, A. 2013. Recent Excavations at Alamgirpur, Meerut District: A Preliminary Report, *Man and Environment* 38.1: 32-54.

[10] Shinde, V., Osada, T., and Kumar, M. 2011. *Excavations at Farmana, District Rohtak, Haryana, India 2006-2008*. Kyoto: Indus Project, Research Institute for Humanity and Nature, Japan.

[11] Weber, S.A., Kashyap, A., & Mounce, L. 2011. Archaeobotany at Farmana: new insights into Harappan plant use strategies. In: Shinde, V., Osada, T., & Kumar, M. (eds), *Excavations at Farmana, District Rohtak, Haryana, India, 2006-8*, Kyoto: Research Institute for Humanity and Nature: 808-825.

[12] Joglekar, P.P., Singh, R.N. and Petrie, C.A. 2017. Faunal Remains from Sampolia Khera (Masudpur I), Haryana, *Indian Journal of Archaeology* 2.1: 25-60.

[13] Joglekar, P., Sharada, C.V. and Abhayan, G.S. 2013. Faunal diversity during the Harappan period in Haryana: A review, *Heritage: Journal of Multidisciplinary Studies in Archaeology* 1: 262–287.

[14] Singh, R.N., Petrie, C.A., Singh, A.K. and Singh, M. 2009. Excavations at Masudpur (Hissar District, Haryana) – 2009: a preliminary report, *Bhāratī* 33: 35-49.

[15] Petrie, C.A., Bates, J., Higham, T. and Singh, R.N. 2016. Feeding ancient cities in South Asia: dating the adoption of rice, millet and tropical pulses in the Indus Civilisation, *Antiquity* 90.354: 1489-1504.

[16] Petrie, C.A., Singh, R.N. and Singh, A.K. 2009. Investigating changing settlement dynamics on the plains: the 2009 survey and excavations at Masudpur (Hissar District, Haryana), *Puratattva* 39: 38-49.

[17] Parikh, D. and Petrie, C.A. 2019. 'We are inheritors of a rural civilisation': rural complexity and the ceramic economy in the Indus Civilisation in northwest India, *World Archaeology* 51.2: 252-272.

[18] Bates, Jennifer. 2016. Social Organisation and Change in Bronze Age South Asia: a Multi-Proxy Approach to Urbanisation, Deurbanisation and Village Life Through Phytolith and Macrobotanical Analysis. PhD thesis, University of Cambridge.

[19] Petrie, C.A. and Bates, J. 2017. 'Multi-cropping', intercropping and adaptation to variable environments in the Indus Civilisation, *Journal of World Prehistory* 30: 81-130.

[20] Joglekar, P.P., Singh, R.N. and Petrie, C.A. 2015. A Preliminary report of animal remains from Bhimwada Jodha (Masudpur VII), Haryana, *Bharati* 39: 1-9.

[21] Trentacoste A., Lightfoot E., le Roux P., Buckley M., Kansa S.W., Esposito C., and Gleba M. 2020. Heading for the hills? A multi-isotope study of sheep management in first millennium BC Italy. *Journal of Archaeological Science: Reports* 29, 102036.

[22] Pin, C., Briot, D., Bassin, C., Poitrasson, F. 1994. Concomitant separation of strontium and samarium-neodymium for isotopic analysis in silicate samples, based on specific extraction chromatography, *Analytica Chimica Acta* 298: 209-217.

[23] Valentine, B., Kamenov, G.D., and Krigbaum, J. 2008. Reconstructing Neolithic groups in Sarawak, Malaysia through lead and strontium isotope analysis. *Journal of Archaeological Science* 35: 1463-1473.

[24] Valentine, B.T. 2013: *Immigrant Identity in the Indus Civilization: A Multi-site Isotopic Mortuary Analysis. PhD* Thesis submitted to the Department of Anthropology, University of Florida.

[25] Valentine, B.T., Kamenov G.D., Kenoyer J.M., Shinde V., Mushrif-Tripathy V., Otarola-Castilo E., and Krigbaum J. 2015. Evidence for patterns of selective urban migration in the Greater Indus Valley (2600–1900 BC): a lead and strontium isotope mortuary analysis *PLoS ONE* 10(4): e0123103.

[26] Kenoyer, J.M., Price, T.C. and Burton, J.H. 2013: A new approach to tracking connections between the Indus Valley and Mesopotamia: Initial results of strontium isotope analyses from Harappa and Ur *Journal of Archaeological Science* 40: 2286–2297.

[27] Chase, B., Meiggs, D., Ajithprasad, P. and Slater, P.A. 2014. Pastoral land-use of the Indus Civilization in Gujarat: faunal analyses and biogenic isotopes at Bagasra *Journal of Archaeological Science* 50: 1–15.

[28] Chakraborty, K.S., Chakraborty, S., le Roux, P, Miller, H.M.-L., Shirvalkar, P. and Rawat, Y. 2018. Enamel isotopic data from the domesticated animals at Kotada Bhadli, Gujarat, reveals specialized animal husbandry during the Indus Civilization *Journal of Archaeological Science: Reports* 21: 183–199.

[29] Chase, B., Meiggs, D. and Ajithprasad, P. 2020. Pastoralism, climate change, and the transformation of the Indus Civilization in Gujarat: Faunal analyses and biogenic isotopes *Journal of Anthropological Archaeology* 59: 101173.

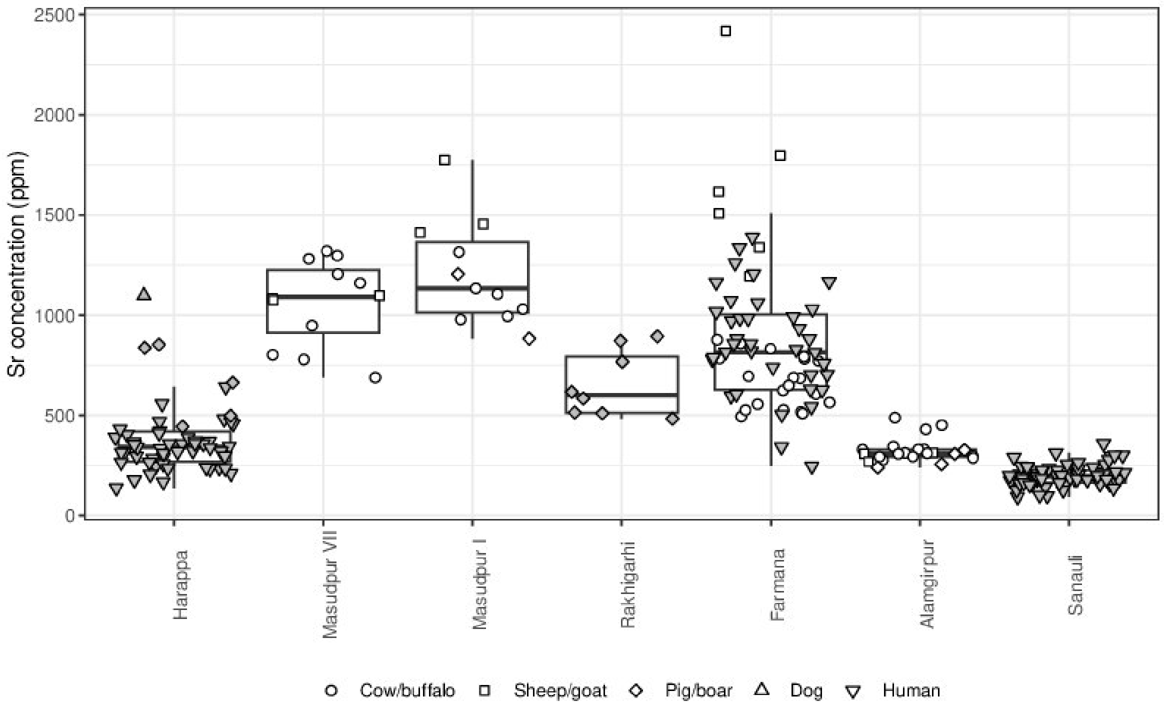

Figure SI 2: Boxplot of animal and human enamel strontium concentration values by site. Empty symbols represent data from this study, grey symbols are data from published research [24] [image generated using R].

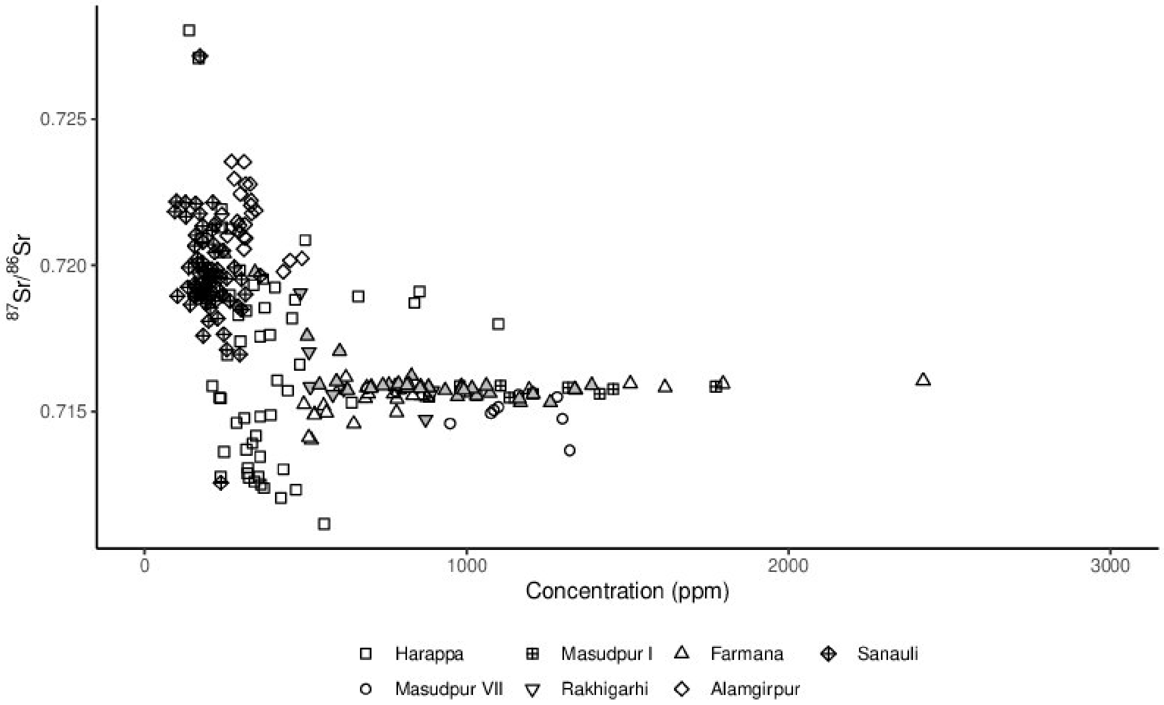

Figure SI 3: Scatterplot of animal and human enamel strontium isotope results against strontium concentration values. Empty symbols represent data from this study, grey symbols are data from published research [24] [image generated using R].

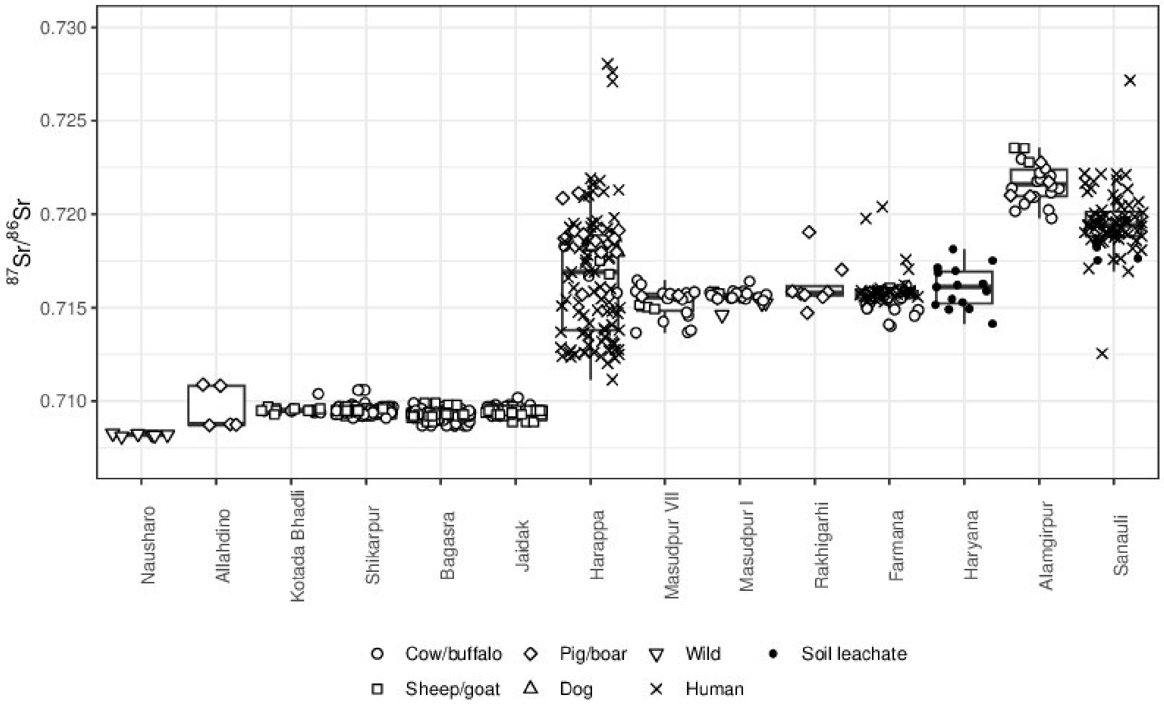

Figure SI 4: Boxplot of animal and human enamel and soil leachate strontium isotope results summarised by site/area from the Indus Civilisation. Data are from this study [24,25,26,27,28,29][image generated using R].

Supplementary Information: Tables

Table SI 1: Soil leachate strontium isotope data from this study.

Soil leachate sample	87/86Sr	±2s internal
STR01	0.715890	0.000013
SRT02	0.714147	0.000013
SRT03	0.715469	0.000011
STR04	0.716209	0.000011
SRT05	0.714902	0.000009
STR06	0.715157	0.000011
SRT07	0.716884	0.000009
STR08	0.714940	0.000015
STR09	0.717521	0.000016
STR10	0.716282	0.000011
STR11	0.716976	0.000012
STR12	0.718137	0.000013
STR13	0.717134	0.000013
STR14	0.715289	0.000017
STR15	0.716102	0.000013

Table SI 2: Complete list of animal samples, sample details and results from this study. Note that the samples analysed at the University of Florida (with BCL numbers, marked by an *) have been adjusted to account for differences in references values (see SI 3 for more information).

Sample	Ind	Site	Tooth	Species	Species2	Period	Cultural Phase	DEJ	Position	Sr	±2s internal	Conc	%RSD	BCL no.	Reference
**FR_1704a**	FR_1704	Farmana	M3	Bos indicus	Cow/buffalo	MHar	Mhar ii	24.43	1	0.715243	0.000013	494.1	0.461		This study
**FR_1704b**	FR_1704	Farmana	M3	Bos indicus	Cow/buffalo	MHar	Mhar ii	15.62	2	0.715192	0.000013	555.4	0.155		This study
**FR_1704c**	FR_1704	Farmana	M3	Bos indicus	Cow/buffalo	MHar	Mhar ii	6.3	3	0.714968	0.000011	782	0.373		This study
**FR_1709a**	FR_1709	Farmana	M3	Bos indicus	Cow/buffalo	MHar	Mhar ii	35.01	1	0.714901	0.000014	526.8	0.722		This study
**FR_1709b**	FR_1709	Farmana	M3	Bos indicus	Cow/buffalo	MHar	Mhar ii	19.5	2	0.714873	0.000011	526.1	0.191		This study
**FR_1709c**	FR_1709	Farmana	M3	Bos indicus	Cow/buffalo	MHar	Mhar ii	4.92	3	0.714958	0.000013	564.3	0.335		This study
**FR_1711_M2a**	FR_1711_M2	Farmana	M2	Bos indicus	Cow/buffalo	MHar	Mhar ii	14.44	1	0.715441	0.000012	685.3	0.535		This study
**FR_1711_M2c**	FR_1711_M2	Farmana	M2	Bos indicus	Cow/buffalo	MHar	Mhar ii	7.03	2	0.715419	0.000009	783.1	0.783		This study
**FR_1711_M3a**	FR_1711_M3	Farmana	M3	Bos indicus	Cow/buffalo	MHar	Mhar ii	21.18	1	0.715593	0.000012	694.6	0.704		This study
**FR_1711_M3b**	FR_1711_M3	Farmana	M3	Bos indicus	Cow/buffalo	MHar	Mhar ii	13.5	2	0.715815	0.000013	605.4	0.921		This study
**FR_1711_M3c**	FR_1711_M3	Farmana	M3	Bos indicus	Cow/buffalo	MHar	Mhar ii	5.63	3	0.71617	0.000011	624.5	0.217		This study
**FR_1713a**	FR_1713	Farmana	M3	Ovis aries	Sheep/goat	MHar	Mhar ii	28.55	1	0.715939	0.000012	1796	0.347		This study
**FR_1713b**	FR_1713	Farmana	M3	Ovis aries	Sheep/goat	MHar	Mhar ii	16.82	2	0.716044	0.000012	2418	0.289		This study
**FR_1713c**	FR_1713	Farmana	M3	Ovis aries	Sheep/goat	MHar	Mhar ii	4.28	3	0.715821	0.000011	1616	0.249		This study
**FR_1717a**	FR_1717	Farmana	M1/M2	Capra/Ovis	Sheep/goat	MHar	Mhar ii	24.16	1	0.715737	0.000012	1338	0.215		This study
**FR_1717b**	FR_1717	Farmana	M1/M2	Capra/Ovis	Sheep/goat	MHar	Mhar ii	17.91	2	0.715739	0.000013	1194	0.328		This study
**FR_1717c**	FR_1717	Farmana	M1/M2	Capra/Ovis	Sheep/goat	MHar	Mhar ii	7.56	3	0.715943	0.000012	1508	0.728		This study
**FR_1720a**	FR_1720	Farmana	M3	Bos indicus	Cow/buffalo	MHar	Mhar ii	30.49	1	0.715815	0.000012	688.8	0.497		This study
**FR_1720b**	FR_1720	Farmana	M3	Bos indicus	Cow/buffalo	MHar	Mhar ii	18.44	2	0.715794	0.000014	792.3	0.116		This study
**FR_1720c**	FR_1720	Farmana	M3	Bos indicus	Cow/buffalo	MHar	Mhar ii	4.87	3	0.71548	0.00001	876.9	0.44		This study
**FR_1729a**	FR_1729	Farmana	M3	Bos indicus	Cow/buffalo	MHar	Mhar ii	49.29	1	0.714018	0.000012	517.2	0.576		This study
**FR_1729b**	FR_1729	Farmana	M3	Bos indicus	Cow/buffalo	MHar	Mhar ii	26.9	2	0.714106	0.000011	509.3	0.743		This study
**FR_1729c**	FR_1729	Farmana	M3	Bos indicus	Cow/buffalo	MHar	Mhar ii	6.28	3	0.714572	0.000013	649.1	0.118		This study
**FR_1731a**	FR_1731	Farmana	M3	Bos indicus	Cow/buffalo	MHar	Mhar ii	52.9	1	0.715541	0.000009	858.7	0.343		This study

Sample	Ind	Site	Tooth	Species	Species2	Period	Cultural Phase	DEJ	Position	Sr	±2s internal	Conc	%RSD	BCL no.	Reference
**FR_1731b**	FR_1731	Farmana	M3	Bos indicus	Cow/buffalo	MHar	Mhar ii	34.66	2	0.715541	0.000013	832	0.404		This study
**FR_1731c**	FR_1731	Farmana	M3	Bos indicus	Cow/buffalo	MHar	Mhar ii	12.56	3	0.71559	0.000012	771.8	0.357		This study
**MSD_15a**	MSD_15	Masudpur VII	M1	Capra hircus	Sheep/goat	MHar	MHar iii	24.04	1	0.7150410	0.000011	1084	0.337		This study
**MSD_15b**	MSD_15	Masudpur VII	M1	Capra hircus	Sheep/goat	MHar	MHar iii	16.29	2	0.7149350	0.000009	1075	0.237		This study
**MSD_15c**	MSD_15	Masudpur VII	M1	Capra hircus	Sheep/goat	MHar	MHar iii	5.45	3	0.7151450	0.000014	1098	0.38		This study
**MSD_177a**	MSD_177	Masudpur VII	M2	Bubalus bubalis	Cow/buffalo	LHar	ELHar	56.52	1	0.7157680	0.000012	801.8	0.448		This study
**MSD_177b**	MSD_177	Masudpur VII	M2	Bubalus bubalis	Cow/buffalo	LHar	ELHar	37.88	2	0.7156850	0.000014	778.4	0.414		This study
**MSD_177c**	MSD_177	Masudpur VII	M2	Bubalus bubalis	Cow/buffalo	LHar	ELHar	22.6	3	0.7157830	0.000011	688.6	0.237		This study
**MSD_5115a**	MSD_5115	Masudpur VII	M3	Bos indicus	Cow/buffalo	LHar	ELHar	44.4	1	0.7154900	0.000012	1281	0.293		This study
**MSD_5115b**	MSD_5115	Masudpur VII	M3	Bos indicus	Cow/buffalo	LHar	ELHar	23.27	2	0.7155680	0.000011	1160	0.261		This study
**MSD_5115c**	MSD_5115	Masudpur VII	M3	Bos indicus	Cow/buffalo	LHar	ELHar	7.36	3	0.7156270	0.000011	1205	0.372		This study
**MSD_5146a**	MSD_5146	Masudpur VII	M2	Bos indicus	Cow/buffalo	LHar	ELHar	20.58	1	0.7145800	0.00001	947.9	0.856		This study
**MSD_5146b**	MSD_5146	Masudpur VII	M2	Bos indicus	Cow/buffalo	LHar	ELHar	13.9	2	0.7147450	0.000012	1297	0.286		This study
**MSD_5146c**	MSD_5146	Masudpur VII	M2	Bos indicus	Cow/buffalo	LHar	ELHar	8.79	3	0.7136620	0.000012	1320	0.919		This study
**MSD_5126***	MSD_5126	Masudpur VII	M3	Bos indicus	Cow/buffalo	LHar	ELHar	NA	1	0.7154451	0.000021	NA	NA	EN-16-3682	This study
**MSD_5126***	MSD_5126	Masudpur VII	M3	Bos indicus	Cow/buffalo	LHar	ELHar	NA	2	0.7157431	0.000027	NA	NA	EN-16-3683	This study
**MSD_5126***	MSD_5126	Masudpur VII	M3	Bos indicus	Cow/buffalo	LHar	ELHar	NA	3	0.7158791	0.000015	NA	NA	EN-16-3684	This study
**MSD_5118***	MSD_5118	Masudpur VII	M3	Bos indicus	Cow/buffalo	LHar	ELHar	NA	1	0.7164781	0.000018	NA	NA	EN-16-3685	This study
**MSD_5118***	MSD_5118	Masudpur VII	M3	Bos indicus	Cow/buffalo	LHar	ELHar	NA	2	0.7162481	0.000014	NA	NA	EN-16-3686	This study
**MSD_5118***	MSD_5118	Masudpur VII	M3	Bos indicus	Cow/buffalo	LHar	ELHar	NA	3	0.7158371	0.000018	NA	NA	EN-16-3687	This study
**MSD_5121***	MSD_5121	Masudpur VII	M2	Bos indicus	Cow/buffalo	LHar	ELHar	NA	1	0.7136861	0.000017	NA	NA	EN-16-3688	This study
**MSD_5121***	MSD_5121	Masudpur VII	M2	Bos indicus	Cow/buffalo	LHar	ELHar	NA	2	0.7137931	0.000015	NA	NA	EN-16-3689	This study
**MSD_5121***	MSD_5121	Masudpur VII	M2	Bos indicus	Cow/buffalo	LHar	ELHar	NA	3	0.7142631	0.000016	NA	NA	EN-16-3690	This study
**MSD_5151***	MSD_5151	Masudpur VII	DM2	Sus scrofa	Pig/boar	LHar	ELHar	NA	1	0.7156691	0.000012	NA	NA	EN-16-3691	This study
**MSD_5114***	MSD_5114	Masudpur VII	M2	Sus scrofa	Pig/boar	LHar	ELHar	NA	1	0.7156131	0.000013	NA	NA	EN-16-3692	This study
**MSD_02a**	MSD_02	Masudpur I	M3	Capra/Ovis	Sheep/goat	MHar	MHar iii	33.79	1	0.7158490	0.000012	1774	0.716		This study
**MSD_02b**	MSD_02	Masudpur I	M3	Capra/Ovis	Sheep/goat	MHar	MHar iii	20.69	2	0.7156010	0.000012	1413	0.416		This study

Sample	Ind	Site	Tooth	Species	Species2	Period	Cultural Phase	DEJ	Position	Sr	±2s internal	Conc	%RSD	BCL no.	Reference
**MSD_02c**	MSD_02	Masudpur I	M3	Capra/Ovis	Sheep/goat	MHar	MHar iii	7.37	3	0.7157600	0.000014	1455	0.223		This study
**MSD_03a**	MSD_03	Masudpur I	M2	Bos indicus	Cow/buffalo	MHar	MHar iii	34.79	1	0.7158600	0.00001	978.2	0.563		This study
**MSD_03b**	MSD_03	Masudpur I	M2	Bos indicus	Cow/buffalo	MHar	MHar iii	23.84	2	0.7158790	0.000011	1105	0.534		This study
**MSD_03c**	MSD_03	Masudpur I	M2	Bos indicus	Cow/buffalo	MHar	MHar iii	11.24	3	0.7158140	0.000012	1315	0.564		This study
**MSD_05a**	MSD_05	Masudpur I	M2	Bos indicus	Cow/buffalo	MHar	MHar iii	24.86	1	0.7156720	0.000012	994.2	0.25		This study
**MSD_05b**	MSD_05	Masudpur I	M2	Bos indicus	Cow/buffalo	MHar	MHar iii	15.77	2	0.7155480	0.000011	1030	0.593		This study
**MSD_05c**	MSD_05	Masudpur I	M2	Bos indicus	Cow/buffalo	MHar	MHar iii	7.06	3	0.7154700	0.000012	1133	0.454		This study
**MSD031BULK**	MSD031	Masudpur I	M1	Sus scrofa	Pig/boar	MHar	MHar iii	NA	1	0.7155960	0.00001	1205	0.226		This study
**MSD125BULK**	MSD125	Masudpur I	M1	Sus domesticus	Pig/boar	MHar	MHar iii	NA	1	0.7155000	0.00001	882.7	0.563		This study
**MSD_101_M3***	MSD_101_M3	Masudpur I	M3	A.cervicapra	Wild	MHar	MHar iii	NA	1	0.7152591	0.000011	NA	NA	EN-16-3669	This study
**MSD_NN012***	MSD_NN012	Masudpur I	M3	A.cervicapra	Wild	MHar	MHar iii	NA	1	0.7146171	0.000012	NA	NA	EN-16-3670	This study
**MSD_089***	MSD_089	Masudpur I	M1/M2	Bos indicus	Cow/buffalo	MHar	MHar iii	NA	1	0.7156961	0.000019	NA	NA	EN-16-3671	This study
**MSD_089***	MSD_089	Masudpur I	M1/M2	Bos indicus	Cow/buffalo	MHar	MHar iii	NA	2	0.7157551	0.000016	NA	NA	EN-16-3672	This study
**MSD_089***	MSD_089	Masudpur I	M1/M2	Bos indicus	Cow/buffalo	MHar	MHar iii	NA	3	0.7157691	0.000018	NA	NA	EN-16-3673	This study
**MSD_077***	MSD_077	Masudpur I	M2	B.tragocamelus	Wild	MHar	MHar iii	NA	1	0.7152281	0.000016	NA	NA	EN-16-3674	This study
**MSD_NN010***	MSD_NN010	Masudpur I	M3	Bos indicus	Cow/buffalo	MHar	MHar iii	NA	1	0.7164121	0.000023	NA	NA	EN-16-3675	This study
**MSD_NN010***	MSD_NN010	Masudpur I	M3	Bos indicus	Cow/buffalo	MHar	MHar iii	NA	2	0.7156721	0.000016	NA	NA	EN-16-3676	This study
**MSD_NN010***	MSD_NN010	Masudpur I	M3	Bos indicus	Cow/buffalo	MHar	MHar iii	NA	3	0.7155141	0.000014	NA	NA	EN-16-3677	This study
**MSD_092***	MSD_092	Masudpur I	M2	Bos indicus	Cow/buffalo	MHar	MHar iii	NA	1	0.7155371	0.000018	NA	NA	EN-16-3678	This study
**MSD_092***	MSD_092	Masudpur I	M2	Bos indicus	Cow/buffalo	MHar	MHar iii	NA	2	0.7154881	0.000019	NA	NA	EN-16-3679	This study
**MSD_092***	MSD_092	Masudpur I	M2	Bos indicus	Cow/buffalo	MHar	MHar iii	NA	3	0.7153841	0.000017	NA	NA	EN-16-3680	This study
**ALM_033a**	ALM_033	Alamgirpur	M2	Bos indicus	Cow/buffalo	MHar	MHar iii	24.43	1	0.719783	0.000011	430.3	0.152		This study
**ALM_033b**	ALM_033	Alamgirpur	M2	Bos indicus	Cow/buffalo	MHar	MHar iii	16.4	2	0.720161	0.00001	450.9	0.291		This study
**ALM_033c**	ALM_033	Alamgirpur	M2	Bos indicus	Cow/buffalo	MHar	MHar iii	6.74	3	0.720233	0.000014	488.1	0.483		This study
**ALM_034a**	ALM_034	Alamgirpur	M2	Bos indicus	Cow/buffalo	MHar	MHar iii	30.99	1	0.721772	0.000012	331.1	0.455		This study
**ALM_034b**	ALM_034	Alamgirpur	M2	Bos indicus	Cow/buffalo	MHar	MHar iii	18.66	2	0.721869	0.000011	344.5	0.733		This study
**ALM_034c**	ALM_034	Alamgirpur	M2	Bos indicus	Cow/buffalo	MHar	MHar iii	6.4	3	0.722049	0.000014	329.5	0.607		This study

Sample	Ind	Site	Tooth	Species	Species2	Period	Cultural Phase	DEJ	Position	Sr	±2s internal	Conc	%RSD	BCL no.	Reference
**ALM_036a**	ALM_036	Alamgirpur	M3	Bos indicus	Cow/buffalo	MHar	MHar iii	24.32	1	0.722207	0.000015	329.9	0.322		This study
**ALM_036b**	ALM_036	Alamgirpur	M3	Bos indicus	Cow/buffalo	MHar	MHar iii	17.93	2	0.722436	0.000012	296.9	0.539		This study
**ALM_036c**	ALM_036	Alamgirpur	M3	Bos indicus	Cow/buffalo	MHar	MHar iii	12.15	3	0.722956	0.000014	277.6	0.127		This study
**ALM_135a**	ALM_135	Alamgirpur	M3	Ovis aries	Sheep/goat	MHar	MHar iii	25.43	1	0.722776	0.000011	312.6	0.404		This study
**ALM_135b**	ALM_135	Alamgirpur	M3	Ovis aries	Sheep/goat	MHar	MHar iii	14.97	2	0.723534	0.00001	307.9	0.597		This study
**ALM_135c**	ALM_135	Alamgirpur	M3	Ovis aries	Sheep/goat	MHar	MHar iii	6.95	3	0.723545	0.000011	268.9	0.595		This study
**ALM_152a**	ALM_152	Alamgirpur	M2	Bos indicus	Cow/buffalo	MHar	MHar iii	39.22	1	0.720922	0.000011	312.4	0.76		This study
**ALM_152b**	ALM_152	Alamgirpur	M2	Bos indicus	Cow/buffalo	MHar	MHar iii	24.01	2	0.720554	0.000014	307.7	0.448		This study
**ALM_152c**	ALM_152	Alamgirpur	M2	Bos indicus	Cow/buffalo	MHar	MHar iii	7.59	3	0.721147	0.000012	292.9	0.546		This study
**ALM_153a**	ALM_153	Alamgirpur	M3	Bos indicus	Cow/buffalo	MHar	MHar iii	38.6	1	0.721392	0.000013	310.7	0.443		This study
**ALM_153b**	ALM_153	Alamgirpur	M3	Bos indicus	Cow/buffalo	MHar	MHar iii	27.35	2	0.721363	0.000013	293.8	0.392		This study
**ALM_153c**	ALM_153	Alamgirpur	M3	Bos indicus	Cow/buffalo	MHar	MHar iii	12.34	3	0.721484	0.000013	287	0.299		This study
**ALM031BULK**	ALM031	Alamgirpur	M1/M2	Sus scrofa	Pig/boar	MHar	MHar iii	NA	1	0.722773	0.00001	326.9	0.536		This study
**ALM102BULK**	ALM102	Alamgirpur	M1	Sus domesticus	Pig/boar	PGW	PGW	NA	1	0.720956	0.000012	307.3	0.416		This study
**ALM114BULK**	ALM114	Alamgirpur	M1	Sus domesticus	Pig/boar	PGW	PGW	NA	1	0.721005	0.000012	255.7	0.163		This study
**ALM126BULK**	ALM126	Alamgirpur	M2	Sus scrofa	Pig/boar	MHar	MHar iii	NA	1	0.721738	0.000013	239.1	0.846		This study

Table SI 3: Summary of animal and human enamel strontium concentration data, by site.

Site	n	Mean	Median	IQR	Minimum	Maximum	Range	Reference
Harappa	54	377	342	150	137	1098	961	[24]
Masudpur VII	12	1061	1091	313	689	1320	631	This study
Masudpur I	11	1208	1133	352	883	1774	891	This study
Rakhigarhi	8	655	600	280	483	894	411	[24]
Farmana	63	874	814	376	246	2418	2172	This study
Alamgirpur	22	323	309	37	239	488	249	This study
Sanauli	66	200	196	61	93	359	266	[24]
